# TRPC5-eNOS Axis Negatively Regulates ATP-Induced Cardiomyocyte Hypertrophy

**DOI:** 10.3389/fphar.2018.00523

**Published:** 2018-05-22

**Authors:** Caroline Sunggip, Kakeru Shimoda, Sayaka Oda, Tomohiro Tanaka, Kazuhiro Nishiyama, Supachoke Mangmool, Akiyuki Nishimura, Takuro Numaga-Tomita, Motohiro Nishida

**Affiliations:** ^1^Division of Cardiocirculatory Signaling, Creative Research Group on Cardiocirculatory Dynamism, Exploratory Research Center on Life and Living Systems, National Institute for Physiological Sciences, National Institutes of Natural Sciences, Okazaki, Japan; ^2^Department of Biomedical Science and Therapeutic, Faculty of Medicine and Health Sciences, Universiti Malaysia Sabah, Kota Kinabalu, Malaysia; ^3^Department of Physiological Sciences, School of Life Sciences, The Graduate University for Advanced Studies (SOKENDAI), Okazaki, Japan; ^4^Department of Translational Pharmaceutical Sciences, Graduate School of Pharmaceutical Sciences, Kyushu University, Fukuoka, Japan; ^5^Department of Pharmacology, Faculty of Pharmacy, Mahidol University, Bangkok, Thailand

**Keywords:** cardiac hypertrophy, TRPC cation channels, nitric oxide synthase, NFAT, adenosine triphosphate

## Abstract

Cardiac hypertrophy, induced by neurohumoral factors, including angiotensin II and endothelin-1, is a major predisposing factor for heart failure. These ligands can induce hypertrophic growth of neonatal rat cardiomyocytes (NRCMs) mainly through Ca^2+^-dependent calcineurin/nuclear factor of activated T cell (NFAT) signaling pathways activated by diacylglycerol-activated transient receptor potential canonical 3 and 6 (TRPC3/6) heteromultimer channels. Although extracellular nucleotide, adenosine 5′-triphosphate (ATP), is also known as most potent Ca^2+^-mobilizing ligand that acts on purinergic receptors, ATP never induces cardiomyocyte hypertrophy. Here we show that ATP-induced production of nitric oxide (NO) negatively regulates hypertrophic signaling mediated by TRPC3/6 channels in NRCMs. Pharmacological inhibition of NO synthase (NOS) potentiated ATP-induced increases in NFAT activity, protein synthesis, and transcriptional activity of brain natriuretic peptide. ATP significantly increased NO production and protein kinase G (PKG) activity compared to angiotensin II and endothelin-1. We found that ATP-induced Ca^2+^ signaling requires inositol 1,4,5-trisphosphate (IP_3_) receptor activation. Interestingly, inhibition of TRPC5, but not TRPC6 attenuated ATP-induced activation of Ca^2+^/NFAT-dependent signaling. As inhibition of TRPC5 attenuates ATP-stimulated NOS activation, these results suggest that NO-cGMP-PKG axis activated by IP_3_-mediated TRPC5 channels underlies negative regulation of TRPC3/6-dependent hypertrophic signaling induced by ATP stimulation.

## Introduction

Myocardial hypertrophy is the major predisposing factor for heart failure, arrhythmia and sudden death (Levy et al., 1990). The initiation of hypertrophic stimuli for myocardial hypertrophy has been described through mechanical stress and neurohumoral mechanism that are associated with the release of factors such as angiotensin II (Ang II), endothelin-1 (ET-1) and norepinephrine(Dorn and Force, 2005; Heineke and Molkentin, 2006). Neurohumoral factors are known to stimulate G protein-coupled receptor (GPCR), leading to activation of phospholipase C (PLC), which in turn generates diacylglycerol (DAG) and inositol 1,4,5-trisphosphate (IP_3_) that are responsible for sustained increase in intracellular Ca^2+^ concentration ([Ca^2+^]_i_) (Berridge et al., 2003). The sustained [Ca^2+^]_i_ increase induces pathological myocardial hypertrophy through activation of calcineurin-nuclear factor of activated T cell (NFAT) signaling pathway (Molkentin et al., 1998; Berridge et al., 2003; Heineke and Molkentin, 2006).

Transient receptor potential canonical (TRPC) proteins are now accepted as molecular entities of receptor-activated cation channels (Molkentin et al., 1998; Clapham et al., 2001; Numaga-Tomita et al., 2017). There are seven mammalian TRPC (TRPC1-7) homologues, and TRPC2/3/6/7 subtypes are directly activated by DAG (Hofmann et al., 1999), while TRPC1/4/5 subtypes are activated by IP_3_-mediated Ca^2+^ release from Ca^2+^ store (Zhu et al., 1996). Studies have consistently shown that particularly DAG-activated TRPC3 and TRPC6 (TRPC3/6) channels function as an important mediator of GPCR-stimulated Ca^2+^ signaling pathway that may participate in pathological cardiac hypertrophy (Kuwahara et al., 2006; Nakayama et al., 2006; Onohara et al., 2006). Inhibition of TRPC3/6 channels have been reported to attenuate heart failure through suppressing myocardial hypertrophy and interstitial fibrosis (Kiyonaka et al., 2009; Kitajima et al., 2011, 2016; Numaga-Tomita et al., 2016).

Extracellular nucleotides, especially adenosine 5′-triphosphate (ATP), has long been recognized as an endogenous ligand to stimulate purinergic signaling (Erlinge and Burnstock, 2008). Evidence is accumulating that ATP and other purine/pyrimidine nucleotides play important roles in cardiovascular physiology and pathophysiology (Erlinge and Burnstock, 2008; Nishimura et al., 2017). Following emerging evidence on the role of ATP in cardiac homeostasis, blood circulatory ATP and its metabolites are now considered as reliable biomarkers for cardiac protection (Yeung, 2013; Zimmermann, 2016). ATP exerts its action in cardiomyocytes mostly through GPCR subtypes called P2Y purinergic receptors (Dubyak, 1991; Zimmermann, 2016). As one of the nucleotides released during cell stress, ATP is known to activate Ca^2+^ signaling pathway to initiate various biological processes. Although ATP activates G_q_- PLC-dependent Ca^2+^ signaling pathway in cardiac cells (Nishida et al., 2011), it has been long obscure why ATP never induces cardiomyocyte hypertrophy (Post et al., 1996).

Paracrine modulation of cardiac excitation-contraction coupling (ECC) has recently become a topic of intense interest. Resident heart endothelial cells are well-known physiological paracrine modulators of cardiac myocyte ECC mainly via nitric oxide (NO) and ATP (Mayourian et al., 2018). We previously reported that ATP has potency to induce NO production leading to heterologous downregulation of Ang II type1 receptors (AT1Rs) and senescence in cardiac cells (Nishida et al., 2011). As NO-mediated protein kinase G (PKG) activation through guanylate cyclase-dependent cyclic guanosine 3′,5′-monophosphate (cGMP) production attenuates TRPC3/6 channel activity and calcineurin-NFAT signaling (Fiedler et al., 2002; Nishida et al., 2010), we hypothesize that NO/cGMP/PKG-mediated suppression of TRPC3/6 channel activity underlies negative regulation of hypertrophic growth of cardiomyocytes induced by ATP stimulation. In this study, we demonstrate that ATP induces IP_3_-dependent Ca^2+^ signaling and NO production in neonatal rat cardiomyocytes (NRCMs), as well as functional coupling between IP_3_-responsive TRPC5 channel and endothelial NOS (eNOS) contributes to negative regulation of hypertrophic signaling induced by ATP stimulation.

## Materials and Methods

### Materials and Cell Cultures

4-{[3′,4′-(methylenedioxy)benzyl]amino}-6-methoxyquinazoline (MQ), KT5823, ionomycin and xestospongin C were purchased from Calbiochem. 8-bromo- cyclic guanosine 3′,5′-monophosphate (8-Br-cGMP), S-nitroso-*N*-acetyl-DL-penicillamine (SNAP), 1-oleoyl-2-acetyl-*sn*-glycerol (OAG), ET-1, U73122, 8-Cyclopentyl-1,3-dipropylxanthine (DPCPX) and 2-Aminoethyl diphenylborinate (2-APB) were from Sigma. Ang II was from Peptide Lab. AR-C 118925XX, UTPγS trisodium salt, SCH58261 and MRS1754 were from Tocris Bioscience. Fura 2-AM and Nω-nitro-L-arginine methyl ester hydrochloride (L-NAME) were from Dojindo. Diaminofluorescein-2 diacetate (DAF-2 DA) was from Molecular Probes. Dulbecco’s modified Eagle’s medium (DMEM) was from Wako. Anti-TRPC5, anti-TRPC6 antibodies were from Alomone. Anti-phospho-eNOS (Ser1177), horseradish peroxidase (HRP)-conjugated anti-rabbit IgG and HRP-conjugated anti-mouse IgG were from Santa Cruz Biotechnology. Anti-eNOS antibody was from BD Biosciences. Plasmids encoding NFAT promoter-dependent luciferase and brain natriuretic peptide (BNP) promoter-dependent luciferase, and dual luciferase reporter assay system were from Promega. Collagenase II was from Worthington. Stealth siRNAs for rat TRPC5 and TRPC6, Lipofectamine 2000, and Lipofectamine RNAiMAX reagent were purchased from Invitrogen.

All protocols using rat pups were reviewed and approved by the ethic committees at National Institutes of Natural Sciences or the Animal Care and Use Committee, Kyushu University, and were performed according to the institutional guidelines concerning the care and handling of experimental animals. NRCMs were prepared from the ventricles of 1–2-day-old SD rats as described (Nishida et al., 2010). The minced left ventricular tissue was pre-digested in 0.05% trypsin-EDTA (Gibco) over night at 4°C and then digested in 1 mg/ml collagenase type 2 (Worthington) in PBS for 30 min at 37°C. The dissociated cells were plated in a 10-cm culture dish and incubated at 37°C in a humidified atmosphere (5% CO_2_, 95% air) for 1 h in DMEM containing 10% FBS and 1% penicillin and streptomycin. Floating cells were collected and plated into gelatin-coated culture dishes at a density of around 1.5 × 10^5^ cells/cm^2^. After 24 h, the culture medium was changed to serum-free DMEM. We confirmed that >90% attached cells were actinin-positive by immunostaining. For TRPC knockdown, NRCMs were transfected with each siRNA (50 nM) using Lipofectamine RNAiMAX for 72 h (Nishida et al., 2010; Kitajima et al., 2016).

### Measurement of Intracellular Ca^2+^ Increases and NO Production

Measurement of intracellular Ca^2+^ increases was performed with Fura 2-AM as previously described (Nishida et al., 2010; Kitajima et al., 2016). After aspirating the culture medium from the dishes and washing the cells with DMEM, freshly prepared Fura 2-AM (1 μM) diluted in DMEM was added to the dishes and incubated for 30 min at 37°C. As to the measurement of NO production, NRCMs were incubated with DAF-2 DA (10 μM) for 20 min. The dye solution was then replaced with HEPES-buffered saline solution (HBSS) containing 140 mM NaCl, 5.6 mM KCl, 10 mM glucose, 10 mM HEPES (pH 7.4), 1 mM MgCl_2_ and 2 mM CaCl_2_. CaCl_2_ was omitted in Ca^2+^-free HBSS. Fura-2 was excited by 340 nm and 380 mm UV wavelength and fluorescence images at the emission wavelength of ≥510 nm were recorded and ratiometrically analyzed using a video image analysis system (Aquacosmos, Hamamatsu). DAF-2 DA was excited by 470 ± 20 nm, and the fluorescence images at the wavelength of 525 ± 20 nm were acquired using fluorescence microscopy (BZ-X710, Keyence). The fluorescence intensity of the digital images were analyzed using Image J software. Fold increases in fluorescence were calculated by subtracting fluorescence intensity before stimulation from that after stimulation, which was subsequently divided by that of no-stimulation.

### Reporter Activity

Measurement of NFAT-dependent luciferase activity and BNP promoter-dependent luciferase activity was performed as described previously (Nishida et al., 2010, 2011).

### Western Blot Analysis

NRCMs (1 × 10^6^ cells) plated on 6-well dishes were directly harvested with 2 × SDS sample buffer (200 μl). After centrifugation, supernatants (20–40 μl) were fractionated by 8% SDS-polyacrylamide gel and then transferred onto polyvinylidene difluoride membrane. The expression and phosphorylation of endogenous TRPC proteins were detected by anti-TRPC6 (dilution rate, 1:1000) and anti-TRPC5 (1:1000) antibodies. We visualized the reactive bands using Western Lightning Plus ECL (PerkinElmer). The optical density of the film was scanned and measured with Scion Image software.

### Hypertrophic Responses of Cardiomyocytes

Measurement of hypertrophic responses was performed by measuring the transcriptional activation of BNP gene as described previously (Onohara et al., 2006). Protein synthesis was measured by [^3^H]leucine incorporation (Onohara et al., 2006; Nishida et al., 2010). After cells were stimulated with agonists for 2 h, [^3^H]leucine (1 μCi/ml) was added to the culture medium and further incubated for 4 h. The incorporated [^3^H]leucine was measured using a liquid scintillation counter.

### Statistical Analysis

Results are presented as the mean ± SEM. Statistical comparisons were made using Student’s *t*-test (for two groups) or analysis of variance followed by Tukey’s *post hoc* test (for multiple groups). Values of *P* < 0.05 were considered significant. We made utmost effort to minimize the replicates of animal experiments according to the ethical guideline of 3R (Replacement, Reduction, Refinement).

## Results

### ATP Increases [Ca^2+^]_i_ and NFAT Activity but Fails to Induce Hypertrophic Growth in NRCMs

**FIGURE 1:**
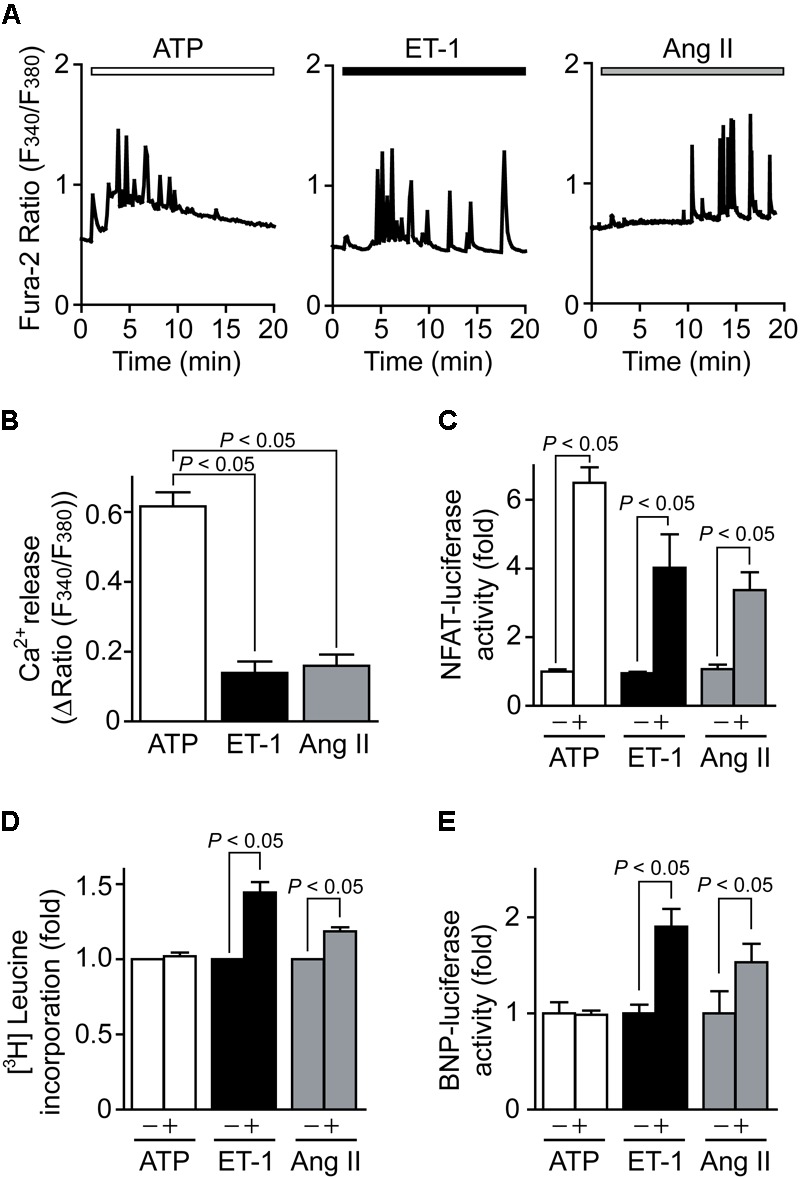
ATP induces Ca^2+^-NFAT signaling but does not induce cardiomyocyte hypertrophy. **(A)** Average time courses of Ca^2+^ response induced by ATP (100 μM), ET-1 (100 nM) or Ang II (1 μM). **(B)** Peak Ca^2+^ increase by agonist stimulation in the absence of extracellular Ca^2+^. *n* = 17–48 cells in each experiment. **(C,D)** Changes in NFAT-dependent reporter activity **(C)** and protein synthesis **(D)** by agonist stimulation. Cells were treated with ATP, ET-1 and Ang II for 6 h. **(E)** Changes in BNP-dependent reporter activities by the stimulation with agonists for 24 h. Error bars indicate SEM.; *n* = 3 **(A–C)**, n = 4 **(D,E)** replicates.

We first investigated the effect of ATP on Ca^2+^-NFAT signaling in NRCMs, as well as known hypertrophy-inducible ligands, ET-1 (Nishida et al., 2010) and Ang II (Onohara et al., 2006). ATP showed repetitive transient [Ca^2+^]_i_ increase events that are superimposed on a substantial maintained increase in [Ca^2+^]_i_, while ET-1 and Ang II induced smaller but sustained oscillatory increases in [Ca^2+^]_i_ (**Figures 1A,B**). ATP increased NFAT-dependent transcriptional activity more potently than ET-1 and Ang II (**Figure 1C**). In contrast, despite increasing NFAT activity, ATP never increased hypertrophic responses, including protein synthesis determined by [^3^H]leucine incorporation and transcriptional activation of BNP (**Figures 1D,E**). These results indicate that ATP-induced increases in [Ca^2+^]_i_ and NFAT activity are not sufficient to induce hypertrophic responses in NRCMs.

### Inhibition of NO Synthesis Potentiates ATP-Induced NFAT Activation and Triggers Hypertrophic Response

**FIGURE 2:**
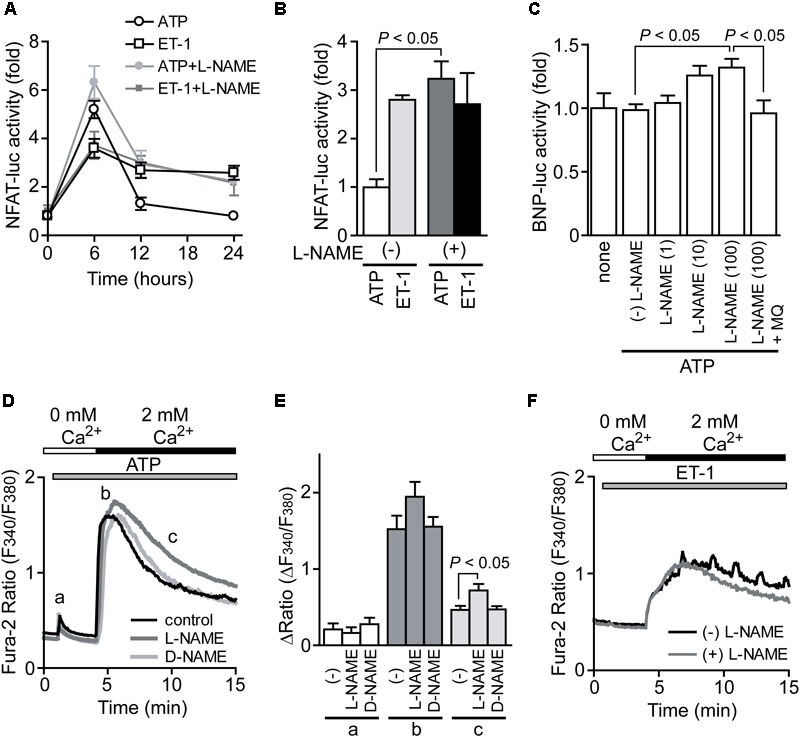
Potentiation of ATP-induced NFAT activation by inhibition of NO synthesis. **(A)** Time courses of NFAT activation by agonist stimulation. Cells were treated with L-NAME (100 μM) 20 min prior to stimulation with ATP (100 μM) or ET-1 (100 nM). **(B)** Effects of L-NAME on the increase in NFAT-luciferase (luc) activities by the stimulation with ATP or ET-1 for 24 h. **(C)** Effects of L-NAME on ATP-induced changes in BNP-luc activities. Cells were treated with indicated concentration (μM) of L-NAME and MQ (10 μM) 20 min prior to ATP stimulation for 24 h. **(D)** Average time courses of ATP-induced Ca^2+^ responses. Ca^2+^ release was first evoked in Ca^2+^-free solution, and Ca^2+^ entry-mediated Ca^2+^ responses were induced by the addition of 2 mM Ca^2+^. Cells were treated with L-NAME and D-NAME (100 μM) 30 min prior to ATP stimulation. **(E)** Peak ATP-induced increases in [Ca^2+^]_i_ in Ca^2+^-free solution (a) and after the addition of Ca^2+^ (b), and [Ca^2+^]_i_ increases sustained 6 min after addition of Ca^2+^ (c). **(F)** Effects of L-NAME on ET-1-induced Ca^2+^ responses. *n* = 22–61 cells. Error bars indicate SEM.; *n* = 8 **(A,B)**, *n* = 4 **(C)**, *n* = 3 **(D–F)** replicates.

We next examined which signal pathway(s) induced by ATP stimulation negatively regulates hypertrophic growth of NRCMs. Previously we reported that mechanical stretch on NRCMs induces ATP release, which leads to NO production through P2Y_2_ receptor stimulation (Nishida et al., 2011). As NO has potent anti-hypertrophic effect by activating cGMP/PKG-dependent pathway in heart (Lefroy et al., 1993), we investigated the involvement of NO in ATP-induced NFAT activation and hypertrophic responses. As shown in **Figures 2A,B**, ATP stimulation induced a powerful transient increase in NFAT activity compared to ET-1 stimulation in NRCMs. Treatment with L-NAME (100 μM), an inhibitor of NOS, resulted in significant enhancement of ATP-induced sustained NFAT activation to the same extent of that by ET-1 stimulation, while L-NAME had no impact on ET-1-induced NFAT activation. L-NAME also enhanced BNP transcriptional activation in response to ATP stimulation (**Figure 2C**). The enhanced BNP activity was canceled by the treatment with MQ (10 μM), a phosphodiesterase (PDE) 5 selective inhibitor, suggesting the involvement of cGMP-dependent pathway. ATP produced a small transient increase in [Ca^2+^]_i_ in the absence of extracellular Ca^2+^ (**Figures 2D,E**, a), which was mainly derived from intracellular IP_3_-responsive Ca^2+^ store. Replenishing extracellular Ca^2+^ led to sustained increases in [Ca^2+^]_i_, which was derived from Ca^2+^ influx probably through store-operated Ca^2+^ channels (**Figures 2D,E**, b and c). Treatment with L-NAME, but not D-NAME (inactive analog of L-NAME), significantly suppressed sustained [Ca^2+^]_i_ increase (**Figures 2D,E**, c). In contrast, although ET-1 stimulation also induced sustained [Ca^2+^]_i_ increase, L-NAME failed to suppress the ET-1-induced sustained [Ca^2+^]_i_ increase in NRCMs (**Figure 2F**). These results suggest that ATP induces NO production in NRCMs and negatively regulates Ca^2+^/NFAT-dependent hypertrophic signaling.

### ATP Activates NO-Dependent Signaling in NRCMs

**FIGURE 3:**
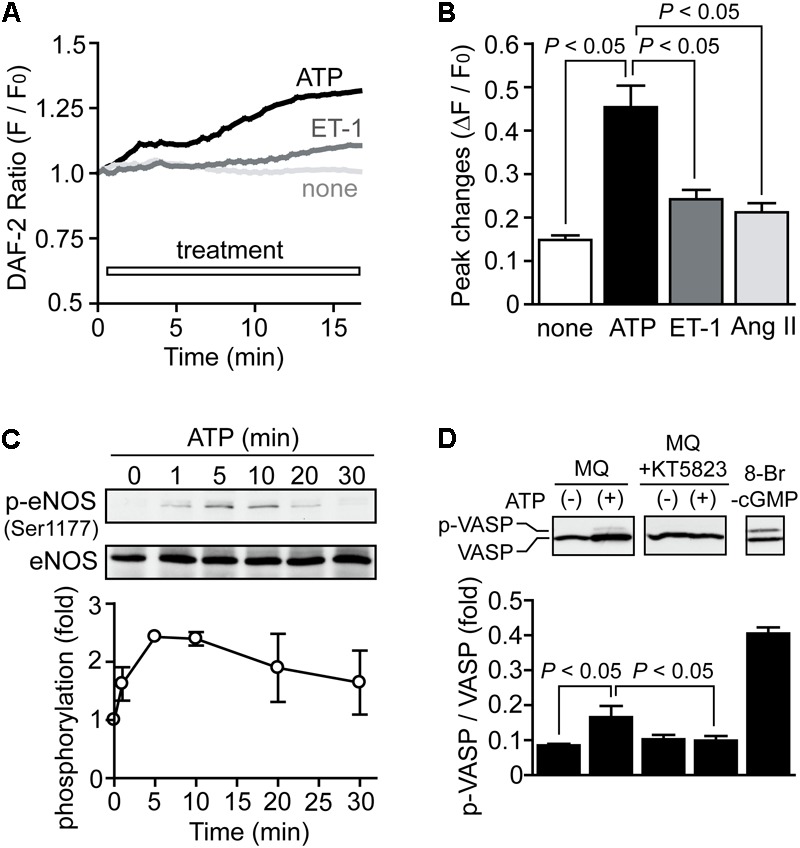
Activation of NO-dependent signaling pathway by ATP. **(A,B)** Average time courses of changes **(A)** and peak increases **(B)** in intracellular NO concentration by agonist stimulation. *n* = 7–22 cells in each experiment. **(C)** Time courses of eNOS phosphorylation induced by ATP. **(D)** PKG-dependent VASP phosphorylation induced by ATP. Cells were treated with MQ (10 μM) and KT5823 (1 μM) 60 min prior to ATP stimulation for 15 min. Cells were also stimulated with 8-Br-cGMP (30 μM) for 15 min as a positive control. Error bars indicate SEM.; *n* = 3 replicates.

We next examined whether ATP actually induces activation of NO-dependent signaling in NRCMs. Compared to stimulation of NRCMs with ET-1 and Ang II, stimulation with ATP significantly increased fluorescence intensity of DAF-2, an NO-sensitive dye (**Figures 3A,B**). Heart expresses all three isoforms of NOS (Balligand and Cannon, 1997), and the activity of the constitutively expressed isoform eNOS is predominantly regulated by its phosphorylation at Ser1177 (Michell et al., 1999). ATP actually increased eNOS phosphorylation at Ser1177 (**Figure 3C**). We further measured the phosphorylation of vasodilator-stimulated phosphoprotein (VASP), a substrate of PKG (Eigenthaler et al., 1992). Stimulation of NRCMs with ATP under PDE5 inhibition slightly but significantly increased phosphorylation level of VASP protein, and this phosphorylation was completely abolished by KT5823 (1 μM), a PKG inhibitor (**Figure 3D**). These results indicate that ATP induces activation of NO-cGMP-PKG pathway in NRCMs.

### TRPC6 Is Not Involved in ATP-Induced Ca^2+^ Responses

**FIGURE 4:**
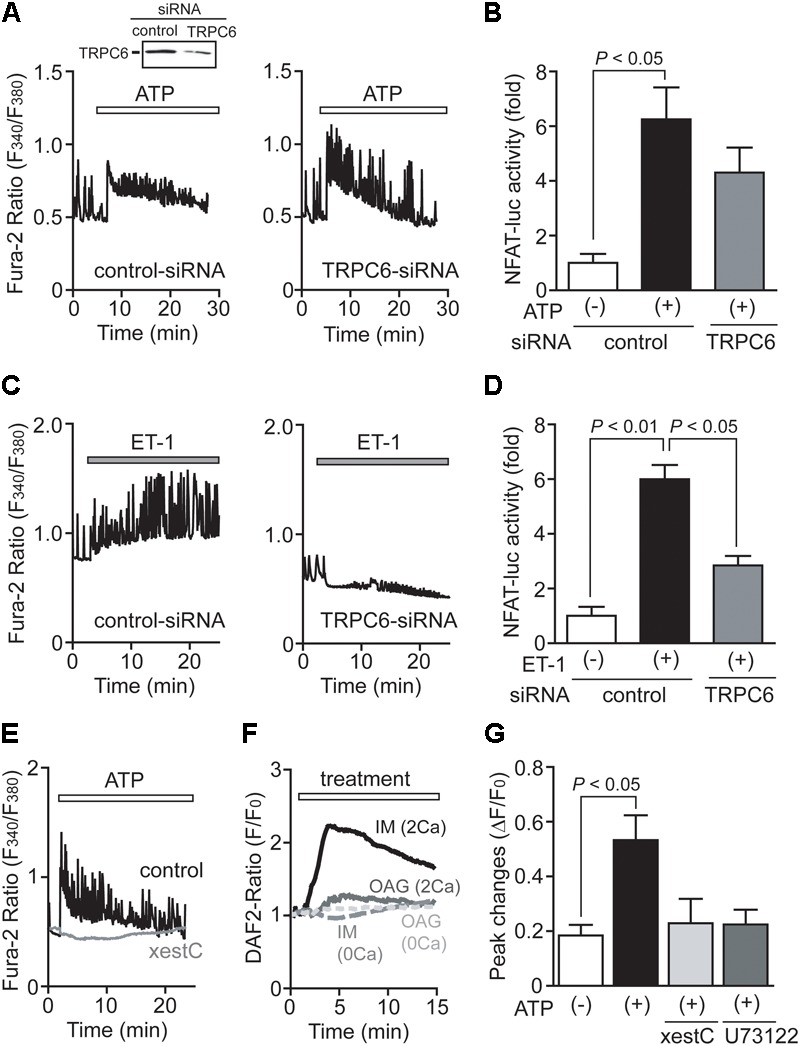
TRPC6 is not involved in ATP-induced Ca^2+^ responses. **(A,C)** Effects of TRPC6-siRNA on Ca^2+^ responses induced by ATP **(A)** or ET-1 **(C)**. *n* = 19 – 32 cells in each experiment. **(B,D)** Effects of TRPC6-siRNA on the increases in NFAT-luciferase activity induced by ATP **(B)** or ET-1 **(D)**. Three days after transfection with TRPC6-siRNA (1786) or control-siRNA, cells were treated with ATP (100 μM) or ET-1 (100 nM) for 6 h. **(E)** Effects of xestospongin C (xestC) on ATP-induced Ca^2+^ responses. *n* = 16–31 cells in each experiment. **(F)** Time courses of changes in intracellular NO concentration induced by ionomycin (IM; 1 μM) or OAG (30 μM) in the presence or absence of extracellular Ca^2+^. *n* = 12 – 38 cells in each experiment. **(G)** Effects of xestC and U73122 on the ATP-induced NO production. Cells were treated with xestC (20 μM) or U73122 (1 μM) 40 min prior to ATP stimulation. Error bars indicate SEM.; *n* = 3 **(A,C,E–G)**, *n* = 4 **(B,D)** replicates.

We previously reported that TRPC6, rather than TRPC3, predominantly mediates mechanical stretch-induced global [Ca^2+^]_i_ increase in NRCMs (Nishida et al., 2010; Kitajima et al., 2016). We also reported that ATP released from NRCMs mediates mechanical stretch-induced G_12/13_ protein signaling in an autocrine/paracrine manner (Nishida et al., 2008). Therefore, we next investigated whether TRPC6 participates in ATP-induced Ca^2+^ signaling in NRCMs. Interestingly, knockdown of TRPC6 failed to attenuate ATP-induced increases in [Ca^2+^]_i_ and NFAT activity in NRCMs (**Figures 4A,B**), while significantly reducing ET-1-induced increases in [Ca^2+^]_i_ and NFAT activity (**Figures 4C,D**). The ATP-induced Ca^2+^ response was completely suppressed by the treatment with xestospongin C (20 μM), an IP_3_ receptor (IP_3_R) blocker (**Figure 4E**). As eNOS activity is predominantly regulated by the increase in [Ca^2+^]_i_ as well as its phosphorylation, we further investigated whether IP_3_-dependent store-operated Ca^2+^ influx signaling mediates eNOS activation by ATP stimulation. Store-operated Ca^2+^ influx was evoked by the treatment with ionomycin (1 μM), a Ca^2+^ ionophore (Nishida et al., 2003). Treatment with ionomycin but not OAG (30 μM), a membrane-permeable DAG analog, increased intracellular NO concentration in the presence of extracellular Ca^2+^ (**Figure 4F**). The ionomycin-induced NO production was completely diminished in the absence of extracellular Ca^2+^, indicating that store-operated Ca^2+^ influx is required for ATP-induced NO production in NRCMs. Additionally, inhibition of IP_3_R or PLC suppressed ATP-induced NO production in NRCMs (**Figure 4G**). These results suggest that ATP-induced Ca^2+^/NFAT signaling and NO production are not mediated by DAG-activated channels, including TRPC6.

### IP_3_-Responsive TRPC5 Channel Partially Participates in ATP-Induced Ca^2+^ Signaling in NRCMs

**FIGURE 5:**
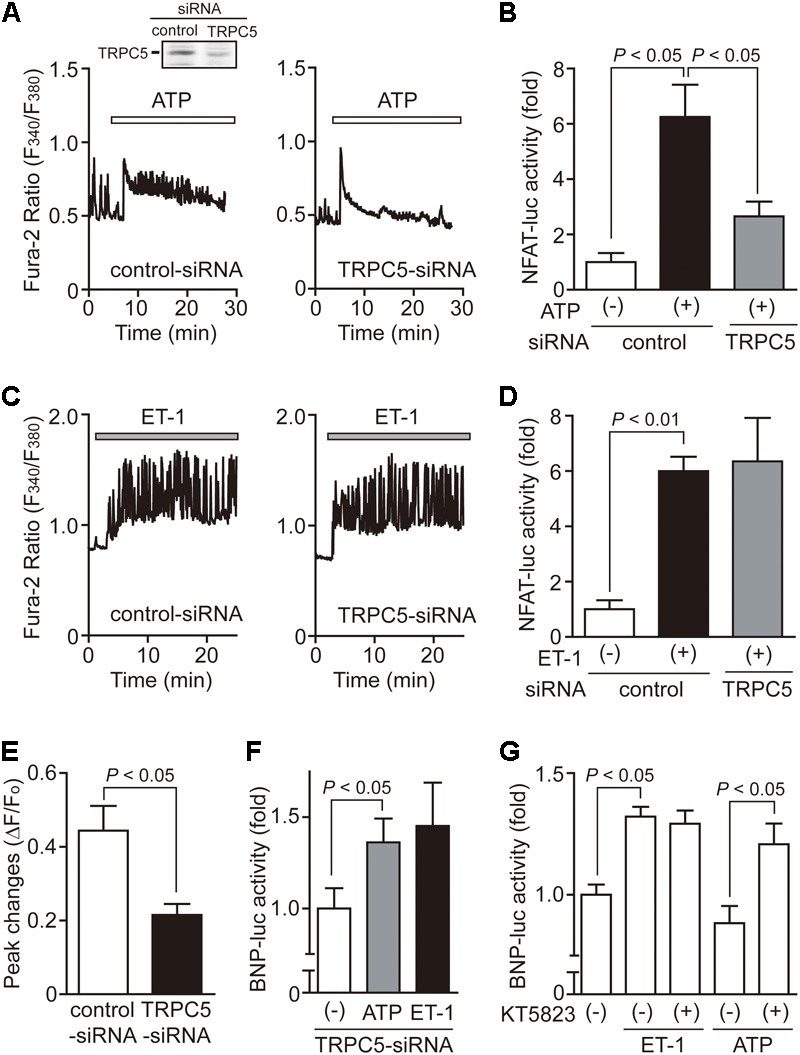
Requirement of TRPC5 in ATP-induced Ca^2+^ responses. **(A,C)** Effects of TRPC5-siRNA on Ca^2+^ responses induced by ATP **(A)** or ET-1 **(C)**. *n* = 19 – 34 cells in each experiment. **(B,D)** Effects of TRPC5-siRNA on the increases in NFAT-luciferase activity induced by ATP **(B)** or ET-1 **(D)**. Three days after transfection with TRPC5-siRNA (276) or control-siRNA, cells were treated with ATP (100 μM) or ET-1 (100 nM) for 6 h. **(E)** Effects of TRPC5 siRNA on ATP-induced NO production. **(F)** Effects of TRPC5-siRNA on the increase in BNP-luciferase activity induced by ATP or ET-1. **(G)** Effects of KT5823 on the increase in BNP-luciferase activity induced by ET-1 or ATP. Cells were treated with KT5823 (1 μM) 60 min prior to agonist stimulation for 24 h. Error bars indicate SEM.; *n* = 3 **(A,C,E)**, *n* = 4 **(B,D,F,G)** replicates.

TRPC5 has been shown to be upregulated in human failing heart, although its physiological role is still not fully understood (Bush et al., 2006). TRPC5 is one of the IP_3_-responsive TRPC channels, and reported to form stable protein complex with eNOS to amplify NO signaling in endothelial cells (Yoshida et al., 2006). We thus investigated whether TRPC5 participates in ATP-induced Ca^2+^ signaling and NO production in NRCMs. Surprisingly, knockdown of TRPC5 significantly suppressed the ATP-induced sustained increase in [Ca^2+^]_i_, but not transient [Ca^2+^]_i_ increase (**Figure 5A**). TRPC5 knockdown also attenuated the NFAT activity in ATP-stimulated NRCMs (**Figure 5B**). In contrast, the ET-1-induced increases in [Ca^2+^]_i_ and NFAT activity were not reduced by TRPC5 knockdown (**Figures 5C,D**). We also found that TRPC5 knockdown markedly reduced ATP-induced NO production (**Figure 5E**), and increased ATP-induced BNP transcriptional activity at a rate similar to ET-1 stimulation (**Figure 5F**). The induction of hypertrophic response by ATP stimulation in TRPC5 knockdown NRCMs were mimicked by the treatment with KT5823 (**Figure 5G**). These results suggest that TRPC5 acts as negative regulator of hypertrophic signaling in NRCMs through eNOS-mediated activation of NO/cGMP/PKG signaling.

### P2Y_2_R-PLC-IP_3_-Ca^2+^ Influx Axis Mediates ATP-Induced NO Production in NRCMs

**FIGURE 6:**
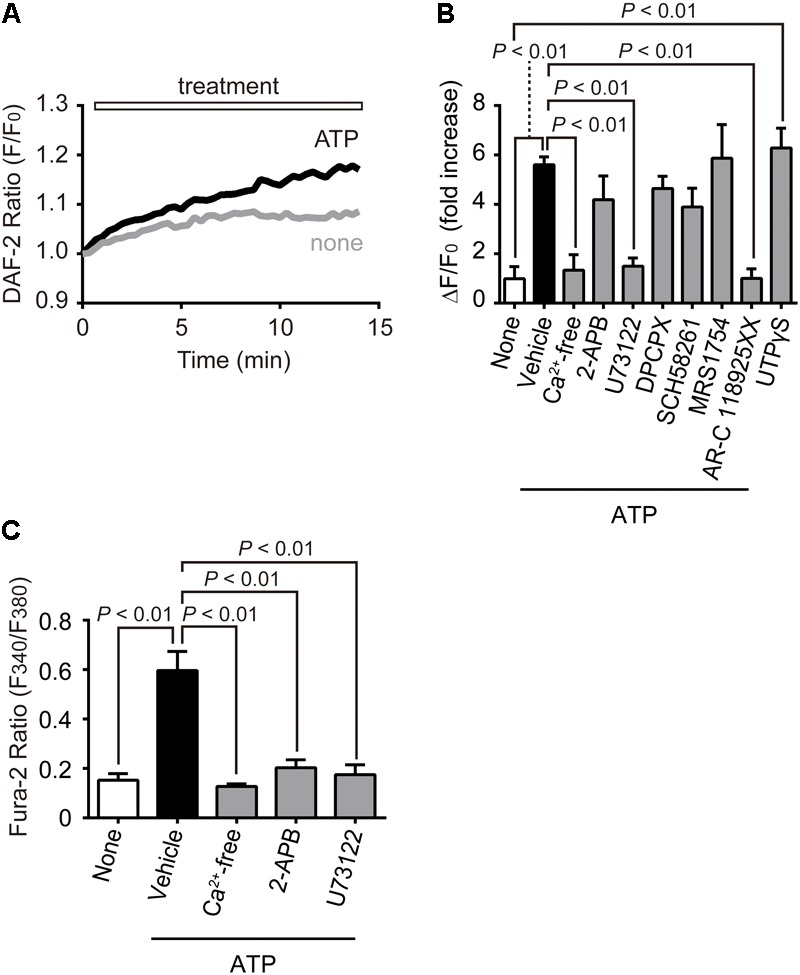
P2Y_2_R-PLC-Ca^2+^ influx axis mediates ATP-induced NO production in NRCMs. **(A)** Time-courses of relative changes of DAF fluorescence intensity (F/F_0_) in the presence or absence of ATP (100 μM). **(B)** Effects of inhibitors on ATP-induced NO production. NRCMs were pretreated with 2-APB (10 μM, *n* = 4 replicates), U73122 (1 μM, *n* = 4), DPCPX (1 μM, *n* = 3), SCH58261 (0.5 μM, *n* = 3), MRS1754 (0.5 μM, *n* = 3) and AR-C118925XX (10 μM, *n* = 3) 20 min before ATP stimulation. NRCMs were stimulated with ATP in the absence of extracellular Ca^2+^ (Ca^2+^-free, *n* = 4). P2Y_2/4_Rs of NRCMs were selectively stimulated with UTPγS (1 μM, *n* = 3). **(C)** Effects of inhibitors on ATP-induced [Ca^2+^]_i_ increases (*n* = 3 replicates). Error bars indicate SEM.

We finally examined which purinergic receptor subtype(s) mediates ATP-induced NO production in NRCMs. As expected, the ATP-induced NO production was significantly suppressed by pharmacological inhibition of PLC by U73122 (1 μM) or P2Y_2_R by AR-C 118925XX (10 μM) (**Figures 6A,B**). Stimulation with UTPγS, a P2Y_2/4_R-selective ligand, also increased NO production. In addition, U73122 suppressed the ATP-induced Ca^2+^ response (**Figure 6C**). As AR-C 118925XX has yellow-colored self-fluorescence that entirely overlaps F340 intensity of Fura-2, we could not measure the exact ratiometric changes of fura-2 induced by ATP stimulation in the presence of AR-C118925XX. However, we confirmed that AR-C 118925XX completely suppressed the ATP-induced increase in F340 intensity of Fura-2 (data not shown). These results strongly suggest that P2Y_2_R-PLC axis predominantly mediates ATP-induced Ca^2+^ response and NO production in NRCMs.

Extracellular ATP is rapidly converted into ADP, AMP and adenosine by membrane-bound ectonucleotidases (Nishimura et al., 2017). Adenosine receptors are reported to participate in the regulation of NO in the heart and many effects of adenosine are mediated via NO-cGMP pathways (Sterin-Borda et al., 2002). However, treatment of NRCMs with DPCPX (1 μM, A_1_R-selective antagonist), SCH58261 (0.5 μM, A_2A_R-selective antagonist), and MRS1754 (0.5 μM, A_2B_R-selective antagonist) did not significantly suppress ATP-induced NO production (**Figure 6**). Therefore, adenosine receptors may hardly contribute to ATP-induced anti-hypertrophic NO signaling in NRCMs.

As the removal of extracellular Ca^2+^ also suppressed ATP-induced Ca^2+^ response and NO production, some Ca^2+^ influx pathway(s) may be activated by ATP stimulation. However, the context of store-operated Ca^2+^ channels (SOCs) are hardly developed in matured cardiomyocytes, and the treatment with 2-APB (10 μM), an inhibitor of SOCs, suppressed Ca^2+^ response but not NO production induced by ATP stimulation. Thus, IP_3_-dependent TRPC5-mediated Ca^2+^ influx pathway, but not other Ca^2+^ release-activated Ca^2+^ influx pathway, is involved in ATP-induced Ca^2+^-dependent NO production in NRCMs.

## Discussion

**FIGURE 7:**
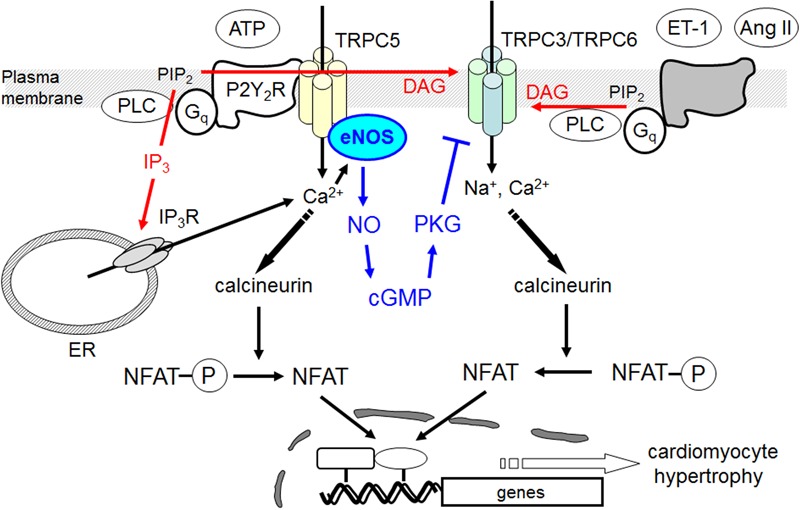
Negative regulation of hypertrophic responses by TRPC5-eNOS signaling axis in cardiomyocyte. TRPC5 functionally couples with eNOS, and activation of TRPC5-mediated NO signaling negatively regulates hypertrophic responses by suppressing TRPC3/C6-mediated Na^+^/Ca^2+^ influx and sustained Ca^2+^/NFAT activation in ATP-stimulated cardiomyocytes.

Although the physiological importance of NO-dependent signaling in heart has been long discussed, the major origin of NO was generally thought to be endothelial cells because of poor expression levels of NOS enzymes in NRCMs. However, local activation of NO signaling in cardiomyocyte induced by sex hormone receptor stimulation has been also attracting attention as a negative regulatory mechanism of cardiac arrhythmia (Bai et al., 2005). The role of Ca^2+^/NFAT signaling in cardiac hypertrophy is well established, and local Ca^2+^ influx through TRPC3/6 channels may be a putative mechanism underlying activation of calcineurin-NFAT signaling pathway in rodent myocardium. We demonstrated that ATP-induced increases in [Ca^2+^]_i_ and NFAT activity are not sufficient to induce hypertrophic responses in NRCMs. Previous studies revealed that sustained Ca^2+^ oscillation is more efficient in activating NFAT-dependent hypertrophic gene expression than transient Ca^2+^ rise induced by hypertrophy-inducible ligands and mechanical stretch (Dolmetsch et al., 1998; Colella et al., 2008). We revealed that ATP stimulation induced a powerful transient [Ca^2+^]_i_ increase compared to other hypertrophy-inducible ligands, Ang II and ET-1. Similarly, although NFAT transcriptional activity was increased transiently, the actual hypertrophic response should require maintained presence of NFAT, which is not achievable with ATP stimulation. A large body of evidence indicated that NO production is likely to represent a protective mechanism against cardiac hypertrophy (Balligand and Cannon, 1997). We found that ATP can induce NO production in NRCMs. Inhibition of NO production by L-NAME indeed resulted in potentiation of ATP-induced sustained NFAT activity and induction of hypertrophic response. ATP acts on both P2X channels and P2Y receptors, and P2Y_2_ receptor is predominantly expressed in NRCMs (Nishida et al., 2008, 2011). We previously reported that P2Y_2_ receptor stimulation induces NO signaling in rat cardiac fibroblasts and NRCMs through induction of inducible NOS (iNOS) (Nishida et al., 2011). In this study, we newly found that TRPC5 participates in ATP-stimulated NO signaling in NRCMs. While it is still unclear whether TRPC5 also participates in ATP-induced iNOS induction of NRCMs, our results clearly suggest that TRPC5-mediated NO signaling contributes to negative regulation of sustained NFAT signaling and hypertrophic responses induced by ATP in NRCMs (**Figure 7**).

Although TRPC5 expression is upregulated in pathologic hypertrophied human hearts (Bush et al., 2006), its physiological meaning has been still obscure. We demonstrated that TRPC5-mediated Ca^2+^ influx negatively regulates ATP-induced hypertrophic response of NRCMs through activation of NO signaling. Indeed, suppression of TRPC5 resulted in reduction of ATP-induced NFAT activation and NO production, thus promotes hypertrophic response in NRCMs. Although NFAT activation has been long associated with hypertrophic gene expression, we speculate that the transient activation of NFAT mediated by TRPC5-dependent Ca^2+^ entry is not sufficient to induce hypertrophic gene expression in NRCMs.

Because of limitation of the study, we could not determine whether the TRPC5-eNOS axis induced by P2Y_2_R stimulation in neonatal cardiomyocytes is also applicable to adult cardiomyocytes. As TRPC5-mediated NO signaling requires IP_3_-mediated Ca^2+^ release and IP_3_-mediated Ca^2+^ signaling is down-regulated in adult cardiomyocytes compared to that in neonatal cardiomyocytes, contribution of TRPC5-eNOS axis might be minor in normal adult cardiomyocytes. Future study using adult cardiomyocytes will be necessary to elucidate the pathophysiological role of TRPC5-eNOS axis in heart.

In summary, we revealed a physiological role of TRPC5 channel in rat cardiomyocytes. TRPC5 functionally couples with eNOS, and activation of TRPC5-mediated NO signaling induced by ATP stimulation negatively regulates Ca^2+^/NFAT-dependent hypertrophic response of NRCMs. Purinergic receptors are well accepted as an attractive therapeutic target of age-related cardiovascular diseases (Sunggip et al., 2017), and we suggest the potential benefits of the use of P2Y_2_R agonists in the prevention of cardiac hypertrophy. Our new finding will provide a new therapeutic strategy for the prevention of pathological cardiac hypertrophy.

## Author Contributions

MN supervised and conceived the project. CS, KS, and SO designed the experiments and prepared the manuscript. CS, KS, SO, TT, KN, and SM performed the experiments and interpreted the data. MN, AN, and TN-T edited the manuscript.

## Conflict of Interest Statement

The authors declare that the research was conducted in the absence of any commercial or financial relationships that could be construed as a potential conflict of interest. The reviewer AA-T and handling Editor declared their shared affiliation.
